# Identification of complex metabolic states in critically injured patients using bioinformatic cluster analysis

**DOI:** 10.1186/cc8864

**Published:** 2010-02-02

**Authors:** Mitchell J Cohen, Adam D Grossman, Diane Morabito, M Margaret Knudson, Atul J Butte, Geoffrey T Manley

**Affiliations:** 1 Department of Surgery, University of California, 505 Parnassus Avenue, San Francisco, CA 94143, USA; 2 Department of Bioengineering, Stanford University, 450 Serra Mall, Stanford, CA 94305, USA; 3 Department of Neurosurgery, University of California, 505 Parnassus Avenue, San Francisco, CA 94143, USA; 4 Departments of Medicine and Pediatrics (Medical Informatics), Stanford University, 450 Serra Mall, Stanford, CA 94305, USA

## Abstract

**Introduction:**

Advances in technology have made extensive monitoring of patient physiology the standard of care in intensive care units (ICUs). While many systems exist to compile these data, there has been no systematic multivariate analysis and categorization across patient physiological data. The sheer volume and complexity of these data make pattern recognition or identification of patient state difficult. Hierarchical cluster analysis allows visualization of high dimensional data and enables pattern recognition and identification of physiologic patient states. We hypothesized that processing of multivariate data using hierarchical clustering techniques would allow identification of otherwise hidden patient physiologic patterns that would be predictive of outcome.

**Methods:**

Multivariate physiologic and ventilator data were collected continuously using a multimodal bioinformatics system in the surgical ICU at San Francisco General Hospital. These data were incorporated with non-continuous data and stored on a server in the ICU. A hierarchical clustering algorithm grouped each minute of data into 1 of 10 clusters. Clusters were correlated with outcome measures including incidence of infection, multiple organ failure (MOF), and mortality.

**Results:**

We identified 10 clusters, which we defined as distinct patient states. While patients transitioned between states, they spent significant amounts of time in each. Clusters were enriched for our outcome measures: 2 of the 10 states were enriched for infection, 6 of 10 were enriched for MOF, and 3 of 10 were enriched for death. Further analysis of correlations between pairs of variables within each cluster reveals significant differences in physiology between clusters.

**Conclusions:**

Here we show for the first time the feasibility of clustering physiological measurements to identify clinically relevant patient states after trauma. These results demonstrate that hierarchical clustering techniques can be useful for visualizing complex multivariate data and may provide new insights for the care of critically injured patients.

## Introduction

The modern intensive care unit (ICU) is awash in a continuous stream of multivariate data produced from multiple monitors, ventilators, laboratory data and medical staff documentation. The dramatic increase in available information has led to an ICU that is very data-rich. The trauma and critical care communities have turned to these monitors and the data they produce to better understand post-injury physiology and guide resuscitation and treatment. Despite the improvements in, and increasing reliance on monitoring technology, these multivariate data (EKG, arterial blood pressure, ventilator information, and so on) are still recorded intermittently in many ICUs, often as infrequently as every hour, onto a paper chart. Even in ICUs where the paper chart has been replaced by a computerized medical record, these systems are not adequate for the tracking and analysis of complex multivariate relationships. Furthermore, this antiquated, non-relational system of data collection and presentation limits our ability to understand the complex relationship between variables and precludes longitudinal analysis of trends and developing patient pathophysiology. This results in care decisions that are too simplistic in nature. Indeed, most often care orders are written to restrict one variable to a given range (that is, give a fluid bolus for a systolic blood pressure <100) resulting in univariate treatment of complex multivariate physiology. A method to visualize and utilize complex multivariate data is needed, with the ultimate goal of identifying predictive patterns to protocolize and guide medical care. New applications of techniques in bioinformatics and data mining have been developed in the disparate fields of high throughput genomics, physics, and business data management that are aimed at dealing with these increasingly large and complex data sets [1,2]. These data-intensive fields apply techniques such as hierarchical clustering, k-means clustering and self-organizing maps to permit pattern recognition in data sets that would otherwise be too complex to visualize. Investigations in genetic research use hierarchical clustering to group gene expression data according to patterns based on deviations from the mean or median. These clusters are then visualized as a heat map and dendrogram to highlight the similarity within clusters. This has led to an improved understanding of complex genomic interactions and the development of new tools for the diagnosis and management of human disease [3]. We sought to apply these techniques to the complex multivariate physiologic data collected from severely injured patients in a modern ICU.

Here we show that these clustering methodologies from bioinformatics are applicable to continuous rapidly changing multivariate physiologic data in critically injured patients, yielding important insight into patient physiology and outcomes. We define that at any time, the *patient state *is made up of a complex pattern of variables that together make up the resuscitative and metabolic milieu. We further hypothesize that these patterns are not easily discernable using traditional clinical measures of physiology. We define 10 patient states by applying hierarchical clustering to our multivariate ICU data. These states were then characterized based on clinical parameters and patient outcome. The states identified by clustering were not obvious by traditional physiological measures, yet they proved to have clinical prognostic value: time spent in some patient states was significantly predictive of subsequent mortality, the development of multiple organ failure, and infection. Furthermore, patients transitioned through multiple states during their ICU stay, reflecting changing post injury physiology and the effect of resuscitation and treatment. Together these findings demonstrate the potential of these techniques to integrate complex information and provide new insights in clinical care.

## Materials and methods

### Data collection

The study was approved by and conducted under supervision of the Committee on Human Research at the University of California San Francisco. Informed consent was obtained from patients or their surrogates per protocol. Physiological data were collected on 17 severely injured poly-trauma patients at one-minute intervals and stored in our Neurotrauma and Critical Care Database using a multimodal bio-informatics system (Aristein Bioinformatics, Palo Alto, CA, USA).

This system integrates continuous data from the bedside patient monitor (heart rate, oxygen saturation and Mean Arterial Pressure (MAP) with ventilator data and tissue oxygen measurements using a date and time stamp. Intermittent laboratory data, medications, and nursing interventions were derived from the computerized nursing documentation system (CareVue, Philips, Amsterdam, The Netherlands) and integrated with continuous data. Data were stored on a dedicated server in the ICU. Clinical blood gas sampling was supplemented for study purposes using a point-of-care analyzer (Opti CCA, Roche, Mannheim, Germany). Plasma lactate levels were measured (Roche Accutrend^® ^Lactate point of care testing system, Mannheim, Germany). Microdialysis of the deltoid muscle was performed as part of a previously reported study [4] using the Licox^® ^Oxygen Catheter (Integra Neurosciences, Plainsboro, NY, USA) to measure the partial pressure of oxygen in the deltoid muscle as continuous surrogate markers for splanchnic perfusion. Catheters and monitoring took place for seven days or until the patient was extubated.

Patients were selected as a sequential convenience sample but all were severely injured patients that required ICU admission and ongoing resuscitation. The patients were followed until discharge or death, and all complications, including infections and organ dysfunction, were documented in the study database. Infectious complications included bacteremia, urinary tract infection, wound infection, fungemia, sepsis, abscess, infected decubitus ulcer, infected hardware, meningitis, and osteomyelitis. The Multiple Organ Failure (MOF) Score was calculated as described by Ciesla et al [5]. The ordinal MOF score was converted to a binary outcome variable with MOF score ≥4 designated as Multiple Organ Failure. Other outcome variables were mortality and infection.

### Hierarchical clustering

A total of 45 variables of physiological, clinical, and treatment data were collected every minute. For the clustering analysis we used only continuous variables for which the data were complete (heart monitor, ventilator, and microdialysis data), resulting in 52,000 points across 14 variables.

The clustering algorithm proceeds in two main steps: pairwise distance calculations and cluster linkage. For distance calculations, we used the standard Euclidean distance between each data point, which is calculated as

with *d*_*i*, *j *_being the distance between observations i and j, n being the number of elements per observation, and *x*_*k*, *i*/*j *_being element k of observation i or j. These distances are calculated for every pair of observations, yielding m*(m-1)/2 distances for m observations.

With a complete enumeration of the pairwise distances between all observations, the linkage algorithm merges the two *closest *clusters into one, where a cluster can also be a single data point. For this analysis, we use the complete linkage method, which defines the distance between each cluster as

with C(A, B) the distance from cluster A to cluster B. The maximum function indicates that we take the cluster distance to be the maximal distance between any two points in the cluster.

### Univariate Linear Classifier

When using multidimensional analysis techniques, it is important to consider whether simpler univariate techniques could produce similar results. Therefore we attempted to train a univariate linear classifier using linear discriminant analysis (LDA) to classify our binary outcome. LDA produced an *a posteriori *that each data point falls under our outcome assignments.

In order to provide the LDA algorithm with the best possible chance of providing equivalent or better performance as the multivariate clustering we only use a single set of data rather than splitting our data into distinct training and test sets - a non-standard method that advantages the univariate method over the multivariate. We used all of the data that were input into the clustering algorithm as input into the LDA algorithm.

### Between-cluster correlation analysis

We next calculated the Pearson correlation coefficients for each pair of variables within the clusters with the highest and lowest probabilities of death. Significance of correlations was determined using both bootstrapping and label shuffling resampling methods (10,000 iterations of each) to obtain a null distribution for the correlation coefficients. We then compared the corresponding correlation coefficients between the two clusters of interest.

## Results

### Demographic data

We enrolled 17 severely injured patients admitted to the Surgical Intensive Care Unit at San Francisco General Hospital, over a 14-month period between May 2004 and June 2005. As detailed in Table 1, our patients were severely injured with an average Injury Severity Score of 28 ± 10, an average ICU stay of 24 days and an average total hospital stay of 40 days. Patients were enrolled upon arrival in the ICU and microdialysis and Licox oxygen catheters were placed in uninjured deltoid muscle to measure tissue metabolism. Standard monitoring was initiated upon admission to the ICU. Because these patients often underwent significant diagnosis and resuscitation in the Emergency Department (ED), imaging procedures in Radiology, or operative procedures in the Operating Room, the mean time to beginning of data collection was 10.3 ± 4.1 hours from hospital admission and 4.2 ± 3.8 hours from ICU admission. Multivariate data were collected for a mean of 67 ± 48 hours. We were able to collect at least 24 hours of data for each patient, while we obtained at least 72 hours of data on 10 of our 17 patients (59%). Of the 17 patients, 47% developed Multiple Organ Failure (MOF), 65% had documented infections, and there was an 18% mortality rate in our cohort based on their entire hospital stay.

**Table 1:** Patient demographics

	Frequency	Percent
**Gender**:		

Female	4	24
Male	13	76

**Outcome:**		

Live	14	82
Die	3	18

**Mechanisms:**		

Gun shot wound	9	47
Pedestrians vs. auto	3	15
Fall/Jump	2	11
Other penetrating	2	11
MV/MC crash	2	11
Bike crash	1	5

**Complications:**		

MOF	8	47
Infections	11	63

	**Mean ± s.d.**	**Range**

Age (years)	38 ± 18	18 to 72
ICU length of stay (days)	24 ± 21	1 to 78
Hospital length of stay (days)	40 ± 42	1 to 172
ISS	28 ± 10	16 to 50

**Injury sites (maximum AIS)**	**Number of patients**	**% patients**

Abdomen	4	21.0
Extremity/pelvis	1	5.3
Thorax	1	5.3
Multiple	13	68.4

### Hierarchical clustering

**Figure 1:**
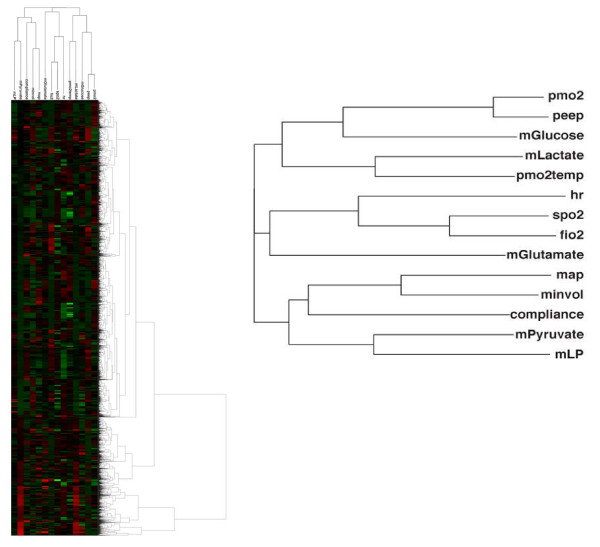
**Heat map and dendrograms for our data set**. In this map, each row represents a row of data (q1 minute) and each column a variable. The color weighting represents normalized levels of each variable from the high (red) to the low (green).

To analyze our multivariate data we used a hierarchical clustering algorithm to place each of the 52,000 minutes of data into 1 of 10 clusters to represent the patient states. The number of clusters was chosen to provide an adequate tradeoff between maximizing intercluster and minimizing intracluster distance. Figure 1 shows the dendrograms for both each minute of data and the physiological variables. To determine if the clustering method was producing physiologically reasonable results and grouping variables that we expect physiologically to group together, we first examined the variable dendrogram (Figure 1) and found that known physiologically related variables were clustered together. For example, SPO_2 _and FIO_2 _are highly physiologically related, and this is represented in their clustering. mPyruvate and mLP clustered together as expected, as mLP is calculated from mPyruvate. Lastly, our group has previously shown that PmO_2 _correlates strongly with increased oxygenation from increasing oxygen delivery [4]. This relationship was manifested in our clustering results with PmO_2 _and PEEP clustering together as well. Because variables we expect to group together actually cluster together, this serves as an internal control of the clustering process and indicates on a gross level that the clustering identifies meaningful groupings of physiology.

### Clinical evaluation of clusters

**Figure 2:**
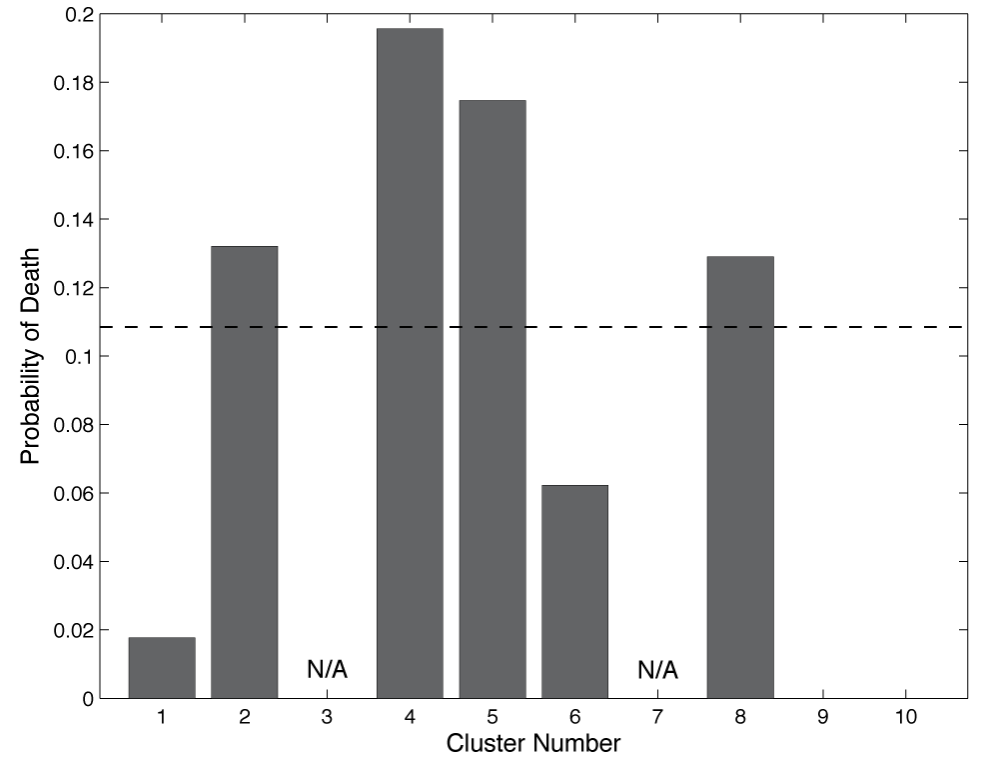
**Probability of death in each cluster**. The baseline death rate (dashed line) is 0.108. Three clusters (2, 4, and 5) had higher representation of physiology correlated with death than. Clusters 3 and 7 had too few data points for the proportions to be meaningful.

**Figure 3:**
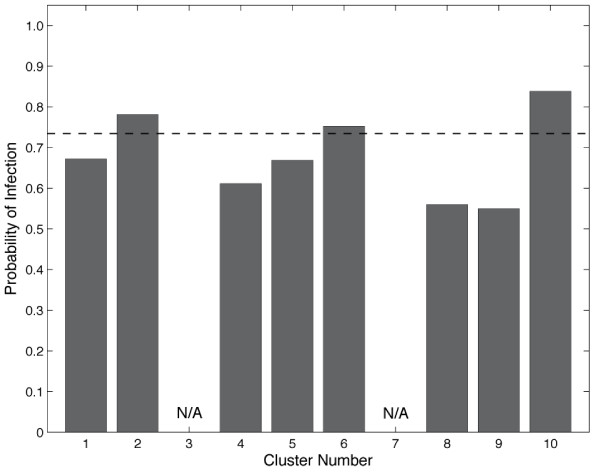
**Probability of infection in each cluster**. The baseline infection rate (dashed line) is 0.735. There were two enriched for infection. Clusters 3 and 7 had too few data points for the proportions to be meaningful.

**Figure 4:**
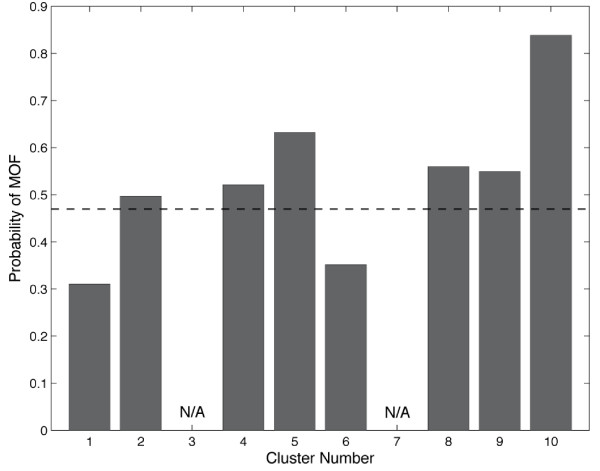
**Probability of multi-organ failure (MOF) in each cluster**. The baseline MOF rate (dashed line) is 0.470. There were six clusters that were enriched for MOF. Clusters 3 and 7 had too few data points for the proportions to be meaningful.

We next examined the states produced from the clustering to determine if any of the clusters represented physiology that would be obvious to an astute clinician. We enumerated the physiological state of each cluster by calculating the means and standard deviations of each of the variables of the clusters (Table 2). Evaluation of the clinical data in these states by four experienced clinicians (intensivists and surgeons) resulted in an inability to clinically define any of the states as sick or well, resuscitated or unresuscitated, and so on, highlighting the difficulty of deriving any traditional clinical prediction or meaning from these patterns. Specifically, none of the clinicians were able to determine whether cluster *x *represented under resuscitation or cluster *y *was that of a well resuscitated patient. Because the clustering method failed to separate the patient data into groups by *obvious *traditional physiological definitions these results confirm our hypothesis that clustering would find meaningful patterns of data that were otherwise impossible to physiologically discern or classify using traditional clinical definitions. We next sought to test the predictive ability of our clustering method by calculating the distribution of patients with particular outcomes across the clusters. This was done for three outcomes: mortality, multiple organ failure (MOF), and infection. Briefly, the percentage of data points in each cluster that were from patients with a given outcome was calculated for each of the three outcomes. A baseline for comparison was calculated by dividing the total number of measurements across the whole data set from patients with a particular outcome by the total number of data points. Figure 2 shows that the baseline number of data points in the entire dataset from patients that died was 10.8%. Three clusters (2, 4, and 5) had higher representation of physiology correlated with death than baseline. Others had an underrepresentation of patients who died (clusters 1, 6, and 10). This was repeated for MOF and infection. Even with increasing baseline values (MOF = 0.47, infection = 0.73) there were six clusters that were enriched for MOF and two enriched for infection (Figures 3 and 4).

**Table 2:** Variable means ± standard deviation for each cluster

	*PERFUSION*				*VENTILATION*					*MICRODIALYSIS*				
	
CLUSTER	MAP	Heart Rate	PmO2	PmO2 Temp	Lung Compliance	PEEP	Minute Vol	SPO2	fiO2	mLactate	mGlucose	mGlutamate	mPyruvate	mLP
Cluster 1	83 ± 13	99 ± 17	37 ± 15	37 ± 1	37 ± 12	8 ± 4	10 ± 2	99 ± 2	52 ± 19	3.7 ± 2.7	7.0 ± 2.3	7.4 ± 5.5	213.2 ± 25.2	0.016 ± 0.011

Cluster 2	82 ± 13	96 ± 21	36 ± 10	37 ± 2	36 ± 14	8 ± 3	10 ± 2	98 ± 2	55 ± 19	2.2 ± 0.7	7.2 ± 2.2	9.2 ± 4.4	113.9 ± 32.7	0.020 ± 0.014

Cluster 3	76 ± 16	102 ± 11	43 ± 13	37 ± 1	199 ± 49	5 ± 0	8 ± 2	100 ± 1	32 ± 5	6.2 ± 1.3	6.6 ± 0.1	5.6 ± 3.3	533.2 ± 28.0	0.012 ± 0.002

Cluster 4	82 ± 12	105 ± 28	31 ± 10	37 ± 1	41 ± 25	7 ± 3	9 ± 2	99 ± 2	45 ± 14	7.3 ± 2.1	6.8 ± 2.2	10.6 ± 15.1	523.4 ± 32.9	0.014 ± 0.004

Cluster 5	87 ± 17	101 ± 26	29 ± 11	36 ± 1	30 ± 12	8 ± 3	9 ± 2	98 ± 4	52 ± 18	7.4 ± 2.0	6.1 ± 2.0	12.4 ± 12.7	430.7 ± 30.0	0.017 ± 0.005

Cluster 6	84 ± 13	104 ± 16	32 ± 11	37 ± 1	34 ± 15	7 ± 3	10 ± 2	99 ± 2	46 ± 15	5.4 ± 2.1	7.0 ± 2.7	12.1 ± 10.5	322.4 ± 39.4	0.017 ± 0.007

Cluster 7	86 ± 2	89 ± 1	45 ± 7	38 ± 0	210 ± 63	4 ± 0	14 ± 1	96 ± 2	40 ± 0	1.1 ± 0.0	7.6 ± 0.1	8.5 ± 0.1	87.2 ± 0.6	0.013 ± 0.000

Cluster 8	81 ± 13	115 ± 11	37 ± 8	37 ± 1	38 ± 23	7 ± 3	9 ± 1	99 ± 1	42 ± 13	10.7 ± 2.7	6.4 ± 2.6	14.6 ± 16.2	697.3 ± 60.0	0.015 ± 0.003

Cluster 9	76 ± 13	109 ± 11	32 ± 8	37 ± 1	34 ± 15	6 ± 1	9 ± 2	99 ± 2	40 ± 9	11.8 ± 2.8	7.9 ± 4.8	11.6 ± 11.3	847.5 ± 51.8	0.013 ± 0.003

Cluster 10	96 ± 25	104 ± 4	23 ± 3	37 ± 1	29 ± 5	5 ± 0	9 ± 2	99 ± 1	41 ± 8	12.3 ± 2.6	11.9 ± 2.9	30.4 ± 26.5	989.5 ± 48.3	0.012 ± 0.003

### Univariate linear classifier

To test whether individual variables were individually statistically significant predictors of outcome we performed Linear Discriminant Analysis (LDA). LDA shows that no single variable was capable of correctly predicting patient outcome significantly better than the chance level of 10.8%. In fact, all but two variables failed to correctly classify a single data point as belonging to a patient who died. The ability of the classifier was poor enough that its optimal strategy was to call every data point as coming from a patient who lived, resulting in an error rate of 10.8%. Even the best classifier (for PmO_2 _Temp) was an inadequate predictor and generated an error rate of 8.5%. This shows that none of the variables are composed of two distinct normally distributed populations with significantly different means and hence are by themselves not predictive of outcome.

### Cluster assignment over time

**Figure 5:**
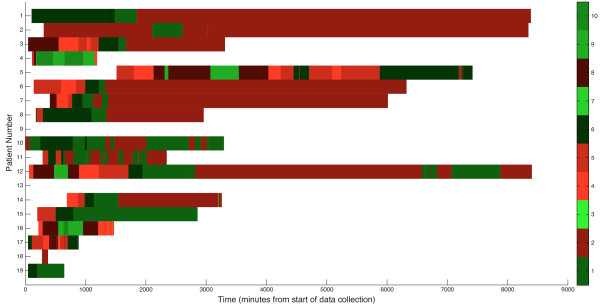
**Assignment of patients to clusters over time**. Cluster numbers are arbitrarily assigned. Brighter shades of red indicate increased probability of death over baseline while brighter shades of green indicate increased probability of life over baseline.

Because we hypothesize that each patient should transition between clusters as physiology and resuscitation state change, we plotted the cluster assignment over time for each patient (Figure 5). Each of the 17 patients spent time in multiple clusters. In addition, each of the three patients who died was in the same cluster (cluster 2) at the end of their monitoring period; one of these patients died at the end of their monitoring period from severe hemorrhagic shock. The other patients who died did so several days to weeks later from multiple organ failure. Despite the discrepancy in the time between the end of monitoring and death, each of these patients was in the same cluster at the end of their monitoring period.

### Cluster representation of novel physiological relationships

**Figure 6:**
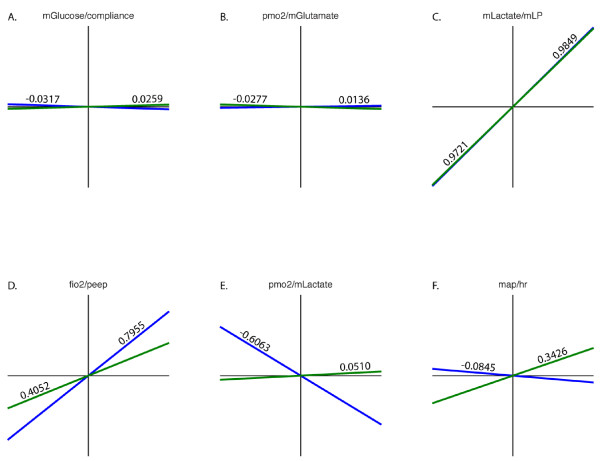
**Correlations of pairs of variables between clusters 1 (live) and 4 (die)**. Cluster 1 is shown in blue and cluster 4 in green. Correlation coefficients are shown on the lines and the variables above each plot.

Having determined that 1) univariate analysis did not provide adequate predictors and 2) that hierarchical clustering provided superior prediction of outcomes, we next sought to determine why this was the case. We hypothesized that the clusters contained new physiological relationships and that the correlations between variable pairs would differ according to patient state. Furthermore, we believed that these changing correlations would likely reflect changing physiological relationships depending on the changing injury or resuscitation state of a patient. To test this we next examined the correlations between pairs of variables within each cluster. To confirm that our correlations were statistically significant, we performed bootstrap resampling and label shuffling. Figure 6 shows the correlation coefficients of variable pairs for cluster 4, the cluster most closely associated with death, and cluster 1, which was most closely associated with good outcome. Examination of these results was very revealing and provided proof of both the discrimination of the clustering technique and the ability of this technique to identify physiologic relationships that would otherwise be impossible to discern.

Several variables showed no correlation or difference between clusters 1 and 4. For example, as expected, compliance and mGlucose were not correlated in either cluster (Figure 6a). This makes physiologic sense as there should be no obvious correlation between these disparate variables, and indeed we can determine no reason a relationship between these variables should be reflected in patient outcome. Furthermore, these two variables were not closely clustered in the physiologic variable dendrogram (Figure 1). Another pair of variables with minimal correlation and no discrimination between outcome clusters is PmO_2 _and mGlutamate (Figure 6b).

Other pairs of correlations represent pertinent physiology that should be similar in patients with any outcome; the strong correlation between mLactate and the ratio between muscle lactate and pyruvate (mLP) is similar in clusters 1 and 4. (Figure 6c). This represents what we know physiologically to be true, namely that as anaerobic respiration takes place there is an increase in both lactate production and pyruvate consumption, resulting in an increase in mLP. Figure 6d also shows a similarly strong positive correlation between FIO2 and PEEP, both variables that clinicians adjust to the degree of physiologic derangement. Close correlation between the variables and similarity between the clusters makes sense, as these parameters are usually adjusted in an identical direction depending on pulmonary physiology.

While these results provide good evidence that the clustering process is physiologically meaningful, we next looked for correlations that were disparate between clusters. Figure 6e shows the correlation between PMO_2 _and mLactate. In cluster 1 there exists the expected correlation of increasing lactate with reduced oxygen. This is in keeping with the relationship between muscle oxygen and lactate that our group has previously described [4]. In the cluster that represented patients who died, however, this basic physiologic effect was lost. Indeed, the correlation between muscle oxygen and lactate was very small, indicating the possibility of cellular or sub cellular (mitochondrial) metabolic dysfunction. Lastly, the opposite direction of the correlations between MAP and HR shown in Figure 6f clearly reflect differences between under resuscitated/critically ill patients and those more likely to survive.

## Discussion

We have shown here the utility of hierarchical clustering as an unsupervised non-linear classification schema in the prediction of outcome in severely injured trauma patients. We obtained clusters that were enriched for patients who died, contracted an infection, and suffered multiple organ failure. These clusters were not merely dominated by a few specific patients with a particular outcome. Indeed each of the clusters was made up of multiple patients' data and each patient transitioned through multiple clusters during their ICU stay. Lastly, the prognostic information incorporated in the clustering results was not obtainable by univariate *traditional statistical *analysis and persists in the face of univariate analyses that could not predict any of these outcomes.

Despite the near continuous monitoring of many physiologic variables and treatment parameters, traditional care in the ICU fails to fully use all these data in an efficient manner. Currently, clinicians base understanding of patient state and appropriate manipulation of that state on intermittent examination of patient variables (vital signs, labs, studies and physical examination). It has been shown, however, that more frequent data collection and analysis better defines patient physiology [6], and there has been much work in using continuous data, including the alarms built into the standard ICU bedside monitors [7,8]. While these monitors are excellent as instant alarms regarding critical parameters, they do nothing to help predict long-term outcomes. Improvements in diagnosis and care have traditionally resulted from both improved clinical acumen and scientific advancement, mostly surrounding scientific examination of a single or small group of adjuncts. Indeed, the critical care literature is full of examinations of monitors, scoring systems, measurements and biomarkers, all of which seek to define and predict the degree of injury, physiological insult and resuscitation [8,9]. Despite this proliferation, multivariate understanding of resuscitation state and identification of occult hypoperfusion remain elusive and an open experimental question. Multivariate decision tools using supervised learning algorithms have been implemented to detect hypovolemia [10] and alarms for critical care patients [8]. In contrast to our current work, this previous work used relatively few types of data (five and nine, respectively), giving a less complete picture of the patient's physiology. Additionally, multiple logistic regression models have been shown to predict MOF 12 hours post-injury [11], but these suffer from the inability to discover new physiology or make use of complex multivariate physiological relationships. In ground breaking work in the mid 90s Rixen and collegues utilized K-means clustering to define patient states based on 17 non-continuous variables. Through clustering and comparison to reference states (derived from non-injured controls) this group elegantly proposed that patient state could be defined in multidimentional state space [12,13]. This work represented the first attempt at defining patient state as a multivariate entity. Here we extend these analyses using continuous data with no a priori understanding of the relationship between these data and outcome. We then extend these analyses by tracing patient state through the *state space *over time.

The use of unsupervised learning with large multivariate data sets comprised of continuous data represents a rarely used combination of techniques to predict and improve patient outcomes. Nelson *et al. *[14] used self-organizing maps to visualize patterns in microdialysis data from patients with traumatic brain injury, finding that individuals were likely to cluster together, in contrast to our results showing much movement among clusters. The work presented here extends previous observations from our group that employed methods similar to those we report here, except that they used aggregate data from each patient rather than q1 minute data, and our methods provide predictions of outcome in addition to the clinical insights discussed by the authors [15]. To fully utilize our data, we required a technique to distill all variables into a meaningful single value - in this case, a patient state. This could then, in turn, be defined in terms of clinically relevant patient outcome or physiologic state, as we have done here by associating each cluster with the probability of an outcome. Instead of fixation on one or a few physiologic parameters, transformation of all data into a single reproducible and clinically relevant value allows all available data to be used simultaneously. Furthermore, the complex relationships among multiple variables are preserved and exploited. Our analysis has shown that without inputting any prior knowledge, unsupervised algorithms are able to discern data (unobtainable by *traditional statistics*) that are indicative of death, infection, and MOF. With our data obtained every minute, the fact that patients transition through many clusters throughout their observation period attests to rapidly changing complex physiology. We have demonstrated our ability to both define patient state using hierarchical clustering and to track the progress of individual patients through these clusters over time. Indeed, patients tend to move between clusters during their stay, and we would expect most of them to experience under-resuscitation during part of their first 24 hours of care. Future analysis could reveal the potential of assigning transition probabilities between clusters based on physiology, which combined with knowledge of the likelihood of death in each state suggests potential methods of *steering *the physiology away from clusters with high mortality towards clusters associated with safety. The ability to do this in real time would greatly improve patient care decisions, leading to potentially enormous gains in outcomes.

We acknowledge that our results are dependent on our choice of similarity measure and clustering method. Our choice of Euclidean distance is natural for the problem at hand, as we were interested in the similarity of all variables to each other, not in how they varied with each other. Though the techniques of *traditional *linear statistics, correlation and regression analyses, can reveal differences between groups or correlations between pairs of physiological variables, we have shown here that they do not easily define a state made up of many variables with complex interrelationships.

There are several limitations to this preliminary study. First, the analysis here is based on a limited number or patients (17) and data points (52,000). Future studies should incorporate more patients (and more data) representing the primary outcomes. While a potential criticism is that a few clusters were dominated by the few patients with poor outcome, resulting in an overfit model, we stress that the clusters were defined in a way blind to patient outcome yet remained enriched for those outcomes.

Our results, while novel, represent a proof of concept study to show that cluster analysis can reveal complex patterns and predict outcome. Even so, we remain aware that to test the general applicability of these results, future studies will have to use a training data set to produce clusters/states that would then be applied to a test data set from separate patients. While we have tried to address the limitations of our single set data and the existence of serial dependence of data points using bootstrap analysis and by showing that each state was populated by data from many patients, future studies can conclusively address these concerns with separate training and test data sets. It also remains unclear how to select the *correct number *of clusters. As there is little guidance in the literature and these analyses have never been attempted in this manner we selected 10 clusters as a trade-off between inter- and intra-cluster distance and a usable number of patient states for analysis. Future studies could easily compare the prognostic information obtained from more or fewer clusters thereby discerning the correct number of states for a similar analysis.

Lastly, while our current work is limited to retrospective assignment of data to clusters, future work should include developing a single score that indicates both the patient's current state and their likelihood of dying during their hospital stay.

## Conclusions

In summary, we have shown the applicability of hierarchical clustering to physiological data to a much greater degree than previous work. We have shown that we can make predictions of outcome and model physiology simultaneously - without specifically including any of our outcome measures in the analysis. Delving into the clustering results enabled us to learn more about the changes in physiology that are more representative of patients dying or living than could be determined using all the data, in aggregate form, from individual patients who lived or died. Comparing correlation coefficients of matching pairs of variables between clusters revealed differences predictive of life and death and disparate physiologic relationships depending on injury and resuscitation state. These insights into physiology also suggest new experiments to determine whether these results hold for larger populations than our polytrauma patients. While preliminary, this analysis shows that complex techniques can improve classification and prediction for severely injured trauma patients. This provides the groundwork for our eventual goal of using automated data-driven methods to provide real time classification and clinical decision support, radically improving outcome for critically ill and injured patients.

## Key messages

• Patient *states *are comprised of complex relationships of constantly changing physiology which are not otherwise discernable to clinicians.

• These states can be defined using ICU data capture and cluster analysis and are enriched for outcomes.

• Patients transition between states based on their injury patterns and resuscitation state.

• Further studies are warranted to explore real time predictive monitoring of patient state, state transition and clinical decision support toward improved outcomes.

## Abbreviations

ED: emergency department; ICU: intensive care unit; LDA: linear discriminate analysis; MAP: mean arterial pressure; MOF: multiple organ failure; PEEP: positive end expiratory pressure; PmO2: partial pressure of muscle oxygen.

## Competing interests

The authors declare that they have no competing interests.

## Authors' contributions

MC collected and processed the data and prepared the manuscript. AG processed the data and prepared the manuscript. GM and DM collected and processed the data and reviewed the manuscript. MMK reviewed the manuscript. AB processed the data and reviewed the manuscript.
